# Incidence, Risk Factors, and Outcomes Associated With Recurrent Neonatal Acute Kidney Injury in the AWAKEN Study

**DOI:** 10.1001/jamanetworkopen.2023.55307

**Published:** 2024-02-08

**Authors:** Austin D. Rutledge, Russell L. Griffin, Katherine Vincent, David J. Askenazi, Jeffrey L. Segar, Juan C. Kupferman, Shantanu Rastogi, David T. Selewski, Heidi J. Steflik

**Affiliations:** 1 Department of Pediatrics, Medical University of South Carolina, Charleston; 2 Department of Epidemiology, University of Alabama at Birmingham; 3 Department of Pediatrics, University of Alabama at Birmingham; 4 Department of Pediatrics, Medical College of Wisconsin, Milwaukee; 5 Department of Pediatrics, Maimonides Medical Center, Brooklyn, New York; 6 Department of Pediatrics, Children’s Hospital at Montefiore, Bronx, New York

## Abstract

**Importance:**

The incidence and associated outcomes of recurrent acute kidney injury (rAKI) in neonates remain largely unknown.

**Objective:**

To determine the incidence, risk factors, and clinical outcomes associated with rAKI in critically ill neonates.

**Design, Setting, and Participants:**

This cohort study was a secondary analysis of the multicenter, international Assessment of Worldwide Acute Kidney Injury Epidemiology in Neonates retrospective study. Comparisons were made among neonates with no AKI, a single AKI episode (sAKI), and rAKI. All neonates younger than 14 days who were admitted between January 1 and March 31, 2014, to 24 participating level II to IV neonatal intensive care units and received intravenous fluids for at least 48 hours were considered for inclusion. Neonates with congenital heart disease requiring surgery within the first week of life, lethal chromosomal anomalies, death within 48 hours of admission, or severe congenital kidney abnormalities were excluded. Data were analyzed from May 23, 2022, to December 8, 2023.

**Exposure:**

Recurrent AKI using the neonatal Kidney Disease: Improving Global Outcomes criteria. Determination of each rAKI required a complete return to the baseline serum creatinine level that defined the prior AKI episode.

**Main Outcomes and Measures:**

Incidence and risk factors of rAKI and associations of rAKI with length of stay (LOS; ie, birth to hospital discharge) and mortality.

**Results:**

The study cohort (n = 2162) included 1233 male neonates (57.0%). Gestational age distribution was less than 29 weeks for 276 neonates (12.8%), 29 to less than 36 weeks for 958 (44.3%), and 36 weeks or older for 928 (42.9%). Of 605 neonates with AKI, 133 (22.0%) developed rAKI with risk factors including younger gestational age, lower birthweight, and higher stage of initial AKI. Infants with rAKI experienced longer median LOS (no AKI, 17 [IQR, 8-34] days; sAKI, 18 [IQR, 9-45] days; rAKI, 60 [IQR, 25-109] days; *P* < .001). Time-varying Cox proportional hazards regression models suggest rAKI is independently associated with a lower hazard of discharge (adjusted hazard ratio, 0.7 [95% CI, 0.6-0.9]; *P* = .01) when compared with sAKI, but mortality did not differ between groups (adjusted hazard ratio, 1.4 [95% CI, 0.6-3.0]; *P* = .44).

**Conclusions and Relevance:**

In this cohort study, neonatal rAKI was independently associated with longer LOS when compared with sAKI, suggesting that rAKI in neonates may be an important clinical distinction warranting further study and careful monitoring after an initial AKI episode.

## Key Points

**Question:**

Are neonates with multiple episodes of acute kidney injury (AKI) at risk for worse outcomes compared with neonates with a single episode?

**Findings:**

In this secondary analysis of a multicenter cohort study including 2162 neonates, recurrent AKI was independently associated with longer length of hospital stay but not increased mortality when compared with those with a single episode.

**Meaning:**

These findings suggest that recurrent AKI in neonates is an important clinical distinction warranting careful monitoring.

## Teaser

This cohort study characterizes the incidence of and risk factors for recurrent neonatal acute kidney injury (AKI) and assesses clinical outcomes associated with recurrent AKI.

## Introduction

Acute kidney injury (AKI) occurs commonly in critically ill neonates and is independently associated with adverse outcomes, including longer length of hospital stay (LOS) and increased mortality.^1,2,3,4,5,6,7,8,9,10^ Acute kidney injury may predispose neonates to subsequent kidney injury, and survivors of neonatal AKI are potentially at risk for long-term complications, including chronic kidney disease.^11,12^ As our understanding of the incidence and impact of neonatal AKI becomes clearer, it is important to better understand the incidence and outcomes associated with the occurrence of more than 1 episode of neonatal AKI after resolution of a previous AKI episode during the same hospitalization, or recurrent AKI (rAKI).

The currently available literature from small, single-center studies suggests an overall rAKI incidence of 7% to 24% (31%-42% of those with any AKI).^13,14,15,16^ Adegboyega et al^13^ found that younger gestational age and lower birthweight are risk factors for rAKI in a cohort of infants with a gestational age less than 28 weeks. In their study, rAKI was associated with a longer LOS and increased mortality when comparing infants with rAKI and those with a single AKI episode (sAKI). In a more recently published single-center study evaluating rAKI in a broader population from a neonatal intensive care unit (NICU) with different gestational ages, Vincent et al^16^ reported that rAKI was associated with longer duration of mechanical ventilation and LOS. However, these findings lack validation by a larger, multicenter study. Though exemplary studies using the international, multicenter Assessment of Worldwide Acute Kidney Injury Epidemiology in Neonates (AWAKEN) cohort have indirectly examined infants with multiple AKI episodes, direct analysis of the incidence, risk factors, and outcomes associated with rAKI has not been performed.^4,17,18^ This knowledge may be used to risk stratify and develop evidence-based post-AKI monitoring guidelines to prevent rAKI.^19,20^

To begin to fill this gap in the literature, we performed a secondary analysis of the multicenter AWAKEN cohort. The aims of this study were to (1) characterize the incidence of rAKI, (2) identify risk factors for rAKI, and (3) determine the association between rAKI and clinical outcomes in neonates. Based on the findings of the previous single-center studies,^13,14,15,16^ we hypothesized that neonates with rAKI would have a younger gestational age and lower birthweight and would experience longer LOS and increased mortality compared with neonates with sAKI.

## Methods

This cohort study followed the Strengthening the Reporting of Observational Studies in Epidemiology (STROBE) reporting guideline. The 24 participating centers received approval from their respective institutional review boards or human research ethics committees. The study design allowed for a waiver of informed consent or parental permission due to retrospective data collection and anonymity of protected health information.

### Study Population

The AWAKEN study is the largest epidemiological evaluation of AKI in neonates to date, with the initial objective of validating the definition of neonatal AKI, identifying risk factors for AKI, and determining whether neonatal AKI is associated with longer LOS and increased mortality. The methodology and protocol for the AWAKEN study have been published previously.^21^ AWAKEN was designed to enrich the cohort with patients at significant risk of AKI while omitting those with limited hospitalization in the NICU. All neonates admitted to level II to IV NICUs from January 1 to March 31, 2014, who were less than 14 days of age and received intravenous fluids for at least 48 hours were considered for inclusion. Exclusion criteria included (1) undergoing surgical repair for congenital heart disease prior to postnatal day 7, (2) lethal chromosomal anomaly, (3) death within 48 hours of admission, and (4) severe, bilateral congenital anomalies of the kidney and urinary tract (such as multicystic dysplastic kidneys, posterior urethral valves, kidney agenesis) (Figure). The decision to exclude neonates who underwent cardiac surgery during the first week of life centered on institution-specific practice patterns regarding postoperative disposition (ie, transfer from the NICU), and this distinct population has been the subject of multiple previous neonatal AKI studies.^2,22,23^

**Figure.**
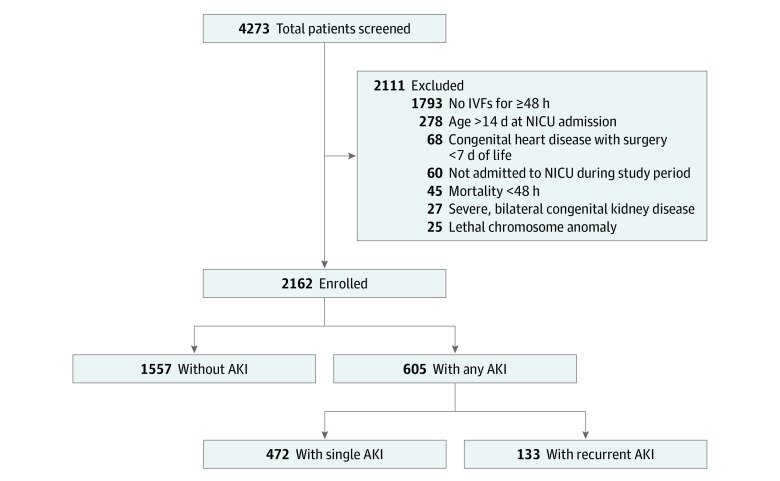
Study Population Flow Diagram AKI indicates acute kidney injury; IVFs, intravenous fluids; and NICU, neonatal intensive care unit.

### Data Collection

A detailed description of the data collection methods for AWAKEN have been published previously.^21^ Trained participants from each of the 24 international centers were responsible for manual medical record review of the participants who contributed data to the AWAKEN cohort. Data from each participant were entered into structured electronic case report forms within the web-based database Rave (Medidata). All data collected pertained to NICU hospitalization following birth. Neonatal and pediatric literature^24,25,26^ suggest racial disparities may affect the incidence and outcomes of AKI and kidney disease; therefore we believed this an important demographic characteristic to include.

### Definition of rAKI

All initial AKI episodes were previously determined in AWAKEN, diagnosed by modified, neonatal Kidney Disease: Improving Global Outcomes (KDIGO) serum creatinine (SCr) or urine output (UOP) criteria.^27^ Recurrent AKI was defined using KDIGO SCr criteria and could only be diagnosed after SCr level returned to at least the lowest baseline value used to diagnose the prior AKI episode during the same hospitalization. For example, if an AKI was associated with an increase in SCr from 0.5 to 1.2 mg/dL, the SCr had to decline to 0.5 mg/dL or less before rAKI could be diagnosed (to convert to μmol/L, multiply by 88.4). A single SCr value that met the KDIGO-defined thresholds for AKI was diagnostic. Urinary output measurements in the AWAKEN dataset were only captured during the first week of life, and availability was institution dependent. Therefore, KDIGO UOP criteria were not included in our rAKI definition. The date of reaching maximum KDIGO staging for any given AKI episode was used for analysis. Two authors (A.D.R. and K.V.) manually reviewed the AWAKEN dataset to identify and stage all rAKI episodes. Any discrepancies were adjudicated by 2 separate authors (D.T.S. and H.J.S.). If an infant died during their index birth hospitalization or was transferred from the NICU or to another institution, data available until death or transfer were reviewed to make AKI determinations.

### Outcomes

The primary outcome was mortality during the index birth hospitalization. The secondary outcome was LOS determined from birth until hospital discharge.

### Statistical Analysis

Data were analyzed from May 23, 2022, to December 8, 2023. Demographic and maternal characteristics during pregnancy and neonatal characteristics at birth, including reasons for admission, were compared among participants with no AKI, sAKI, and rAKI using χ^2^ or Fisher exact tests as appropriate for categorical variables and analysis of variance or Kruskal-Wallis tests for continuous variables. Time-varying Cox proportional hazards regression models (stratified by study center) were used to evaluate, from birth through hospital discharge, the associations between rAKI and LOS as well as rAKI and mortality, and results were expressed as estimated hazard ratios (HRs) with associated 95% CIs. For the LOS model, the event was hospital discharge. For the mortality model, discharge was a censored event. Inclusion of AKI as a time-varying covariate allowed participants to contribute follow-up time to multiple AKI exposure categories (ie, no AKI, sAKI, and rAKI). Two sets of adjusted Cox proportional hazards regression models were created: (1) a model adjusting for gestational age, birthweight, and 5-minute Apgar score and (2) a model built on the first model that used a best-subset process based on Akaike information criterion score to determine additional confounders to include.^28^

In a secondary analysis, interactions between AKI status and gestational age were included in regression models for the estimation of strata-specific associations by gestational age (<29, 29 to <36, and ≥36 weeks) to assess potential effect modification. A Kaplan-Meier curve was used to demonstrate survival over time by AKI status; due to the time-varying nature of the AKI exposure, comparisons of the survival times using either a log-rank or Wilcoxon rank sum test were not performed.

All statistical analyses were performed using SAS, version 9.4 (SAS Institute Inc). A priori, the statistical significance value was determined to be at the level of α = .05.

## Results

Of the 4273 neonates screened, 2162 met the inclusion and exclusion criteria for enrollment and were included in this analysis (Figure). In total, 605 neonates (28.0%) had at least 1 episode of AKI: 472 (78.0%) had sAKI and 133 (22.0%) had rAKI. In the entire cohort, 1233 neonates (57.0%) were boys and 929 (43.0%) were girls. In terms of race and ethnicity, 413 (19.1%) were Black, 293 (13.6%) were Hispanic, 1212 (56.1%) were White, and 537 (24.8%) were of other race or ethnicity. The distribution by gestational age was 276 (12.8%) for neonates aged less than 29 weeks, 958 (44.3%) for those aged 29 to less than 36 weeks, and 928 (42.9%) for those aged 36 weeks or more. Neonates classified as having very or extremely low birthweight comprised 537 (24.8%) of the cohort, and 83 neonates (3.8%) died during NICU hospitalization (Table 1).

**Table 1.** Maternal and Neonatal Characteristics by AKI Status

Characteristic	AKI status^a^			*P* value^b^
	No AKI (n = 1557)	Single AKI (n = 472)	Recurrent AKI (n = 133)	
**Infants**				
Sex				
Boys	883 (56.7)	275 (58.3)	75 (56.4)	.83
Girls	674 (43.3)	197 (41.7)	58 (43.6)	
Ethnicity				
Hispanic	233 (15.0)	43 (9.1)	17 (12.8)	<.001
Non-Hispanic	1084 (69.6)	361 (76.5)	80 (60.2)	
Unknown	240 (15.4)	68 (14.4)	36 (27.1)	
Race				
Black	306 (19.7)	81 (17.2)	26 (19.5)	
White	848 (54.5)	285 (60.4)	79 (59.4)	.18
Other	403 (25.9)	106 (22.5)	28 (21.1)	
Gestational age, wk				
22-28^c^	145 (9.3)	85 (18.0)	46 (34.6)	<.001
29 to <36^c^	790 (50.7)	139 (29.4)	29 (21.8)	
≥36	622 (39.9)	248 (52.5)	58 (43.6)	
Birthweight, g^d^				
≤1000	115 (7.4)	72 (15.3)	47 (35.6)	<.001
1001-1500	246 (15.8)	47 (10.0)	10 (7.6)	
1501-2500	583 (37.5)	99 (21.1)	25 (18.9)	
>2500	610 (39.3)	252 (53.6)	50 (37.9)	
Median 1-min Apgar score (IQR)	7 (5-8)	7 (4-8)	4 (2-7)	<.001
Median 5-min Apgar score (IQR)	8 (7-9)	8 (6-9)	7 (5-8)	<.001
Reasons for admission^e^				
Prematurity <35 wk	823 (52.9)	193 (40.9)	70 (52.6)	<.001
Respiratory symptoms	357 (22.9)	116 (24.6)	34 (25.6)	.64
Respiratory failure	674 (43.3)	203 (43.0)	78 (58.6)	.002
Sepsis evaluation	788 (50.6)	205 (43.4)	69 (51.9)	.02
HIE	75 (4.8)	40 (8.5)	8 (6.0)	.01
Seizures	36 (2.3)	32 (6.8)	5 (3.8)	<.001
Hypoglycemia	192 (12.3)	42 (8.9)	8 (6.0)	.02
Hyperbilirubinemia	38 (2.4)	27 (5.7)	2 (1.5)	.001
Metabolic evaluation	8 (0.5)	10 (2.1)	2 (1.5)	.005
Trisomy 21	14 (0.9)	5 (1.1)	3 (2.3)	.33
Congenital heart disease	43 (2.8)	30 (6.4)	18 (13.5)	<.001
Necrotizing enterocolitis	6 (0.4)	4 (0.8)	11 (8.3)	<.001
Omphalocele and/or gastroschisis	32 (2.1)	10 (2.1)	5 (3.8)	.43
Need for surgical evaluation	48 (3.1)	31 (6.6)	17 (12.8)	<.001
Meningomyelocele	9 (0.6)	6 (1.3)	2 (1.5)	.21
Size for gestational age^d^				
Small	332 (21.4)	85 (18.1)	32 (24.2)	.07
Normal	1150 (74.0)	350 (74.5)	95 (72.0)	
Large	72 (4.6)	35 (7.4)	5 (3.8)	
**Maternal variables**				
Maternal age, mean (SD), y	28.8 (6.2)	28.3 (5.9)	28.0 (6.0)	.21
Pregnancy complications				
Bacterial infection	135 (8.7)	48 (10.2)	18 (13.5)	.14
Viral infection	38 (2.4)	18 (3.8)	5 (3.8)	.23
Diabetes	227 (14.6)	58 (12.3)	10 (7.5)	.05
Hypothyroidism	74 (4.8)	30 (6.4)	3 (2.3)	.13
Chronic hypertension	153 (9.8)	27 (5.7)	11 (8.3)	.02
Kidney disease	13 (0.8)	1 (0.2)	5 (3.8)	<.001
Preeclampsia	242 (15.5)	52 (11.0)	15 (11.3)	.03
Eclampsia	17 (1.1)	7 (1.5)	1 (0.8)	.71
IUGR	147 (9.4)	37 (7.8)	15 (11.3)	.40
Amniotic fluid level				
Oligohydramnios	69 (4.4)	22 (4.7)	11 (8.3)	.005
Normohydramnios	1442 (92.6)	426 (90.3)	112 (84.2)	
Polyhydramnios	46 (3.0)	24 (5.1)	10 (7.5)	
Hemorrhage	43 (2.8)	19 (4.0)	4 (3.0)	.38
Multiple gestation	310 (19.9)	57 (12.1)	10 (7.5)	<.001
Assisted conception	121 (7.8)	29 (6.1)	8 (6.0)	.04
Drugs used during pregnancy				
Corticosteroids	586 (37.6)	126 (26.7)	47 (35.3)	<.001
NSAIDs	48 (3.1)	11 (2.3)	5 (3.8)	.60
Antihypertensives	184 (11.8)	30 (6.4)	15 (11.3)	.003
Illicit drugs	123 (7.9)	40 (8.5)	8 (6.0)	.60
Tobacco	161 (10.3)	54 (11.4)	10 (7.5)	.42
Alcohol	28 (1.8)	5 (1.1)	4 (3.0)	.27
SSRIs	43 (2.8)	13 (2.8)	4 (3.0)	.99
Intrapartum complications				
Nuchal cord	94 (6.0)	31 (6.6)	9 (6.8)	.88
Meconium	164 (10.5)	60 (12.7)	14 (10.5)	.41
Severe vaginal bleeding	58 (3.7)	24 (5.1)	9 (6.8)	.14
Shoulder dystocia	14 (0.9)	6 (1.3)	1 (0.8)	.74
Disposition				
Discharged within 120 d	1263 (81.1)	335 (71.0)	65 (48.9)	<.001
Still in NICU at ≥120 d	28 (1.8)	25 (5.3)	28 (21.1)	
Transfer convalescent care	227 (14.6)	55 (11.7)	18 (13.5)	
Transfer escalated care	15 (1.0)	14 (3.0)	6 (4.5)	
Died in hospital	24 (1.5)	43 (9.1)	16 (12.0)	
Median LOS (IQR), d	17 (8-34)	18 (9-45)	60 (25-109)	<.001

Abbreviations: AKI, acute kidney injury; HIE, hypoxic ischemic encephalopathy; IUGR, intrauterine growth restriction; LOS, length of stay; NICU, neonatal intensive care unit; NSAID, nonsteroidal anti-inflammatory drug; SSRI, selective serotonin reuptake inhibitor.

^a^ Unless otherwise indicated, data are expressed as No. (%) of patients. Percentages are rounded and may not total 100.

^b^ Estimated from χ^2^ test for categorical variables and from analysis of variance or Kruskal-Wallis test for continuous variables.

^c^ Upper limit of range includes 6 days.

^d^ Row categories may not sum to column totals due to missing values.

^e^ More than 1 reason may apply.

In all 605 neonates with AKI, the initial episode of AKI was diagnosed as follows: 324 (53.6%) by SCr criteria, 225 (37.2%) by UOP criteria, and 56 (9.3%) by both SCr and UOP criteria. All rAKI episodes (n = 231) were diagnosed by SCr criteria only. Of those with rAKI, the total number of AKI episodes ranged from 2 to 11, with a median of 1 (IQR, 1-2) rAKI episodes per patient. There was no difference in the median time to the initial AKI for those with sAKI compared with rAKI (3 [IQR, 2-7] vs 4 [IQR, 2-11] days; *P* = .10) (Table 2). In the 133 neonates with rAKI, the second AKI episode (ie, the first rAKI) occurred at a median of 18 (IQR, 9-39) days of life.

**Table 2.** Timing and Severity of AKI

Variable	sAKI (n = 472)	rAKI (n = 133)	*P* value^a^
Time to initial AKI, median (IQR), d	3 (2-7)	4 (2-11)	.10
Time to first rAKI, median (IQR), d	NA	18 (9-39)	NA
Initial AKI stage, No. (%)			
1	262 (55.5)	58 (43.6)	.02
2 or 3	210 (44.5)	75 (56.4)	

Abbreviations: AKI, acute kidney injury; NA, not applicable; rAKI, recurrent AKI; sAKI, single AKI.

^a^ *P* values estimated from a Wilcoxon rank sum test for continuous variables and a χ^2^ test for categorical variables.

### Risk Factors Associated With rAKI

Demographic and baseline characteristics by AKI status are depicted in Table 1. Compared with neonates with sAKI, a higher incidence of rAKI was seen among neonates with the youngest gestational age (85 [18.0%] vs 46 [34.6%]; *P* < .001) and lowest birthweight (72 [15.3%] vs 47 [35.6%]; *P* < .001). Recurrent AKI also occured more frequently in those with a more severe (stage 2 or 3) initial AKI episode (210 [44.5%] vs 75 [56.4%]) and lower Apgar scores (median, 7 [IQR, 4-8] vs 4 [IQR, 2-7] for 1 minute and 8 [IQR, 6-9] vs 7 [IQR, 5-8] for 5 minutes) and in those requiring NICU admission for respiratory failure (203 [43.0%] vs 78 [58.6%]), sepsis evaluation (205 [43.4%] vs 69 [51.9%]), congenital heart disease (30 [6.4%] vs 18 [13.5%]), and surgical evaluation (31 [6.6%] vs 17 [12.8%]) (*P* < .05 for all comparisons).

### rAKI Outcomes: Associations With LOS and Hazard of Discharge

When examining LOS among the overall cohort, infants with sAKI and rAKI experienced significantly longer median LOS (18 [IQR, 9-45] and 60 [IQR, 25-109] days, respectively) compared with infants with no AKI (17 [8-34] days; *P* < .001) (Table 1). When evaluating LOS using the hazard of discharge with the best-fit time-varying Cox proportional hazards regression models, rAKI remained independently associated with a lower hazard of discharge and thus longer LOS (adjusted HR [AHR], 0.7 [95% CI, 0.6-0.9]; *P* = .01) when compared with those with sAKI, even after adjusting for multiple potential confounders.

To better understand this association, AKI status and the hazard of discharge were examined by gestational age. No associations between rAKI and LOS were found in infants with gestational age less than 29 weeks or 29 to less than 36 weeks when compared to those with sAKI (Table 3). In contrast, after adjustment for potential confounders, rAKI was associated with significantly longer LOS compared to those with sAKI (AHR, 0.6 [95% CI, 0.4-0.8]; *P* = .002) in the group with gestational age of at least 36 weeks.

**Table 3.** Associations Between Time-Varying AKI Status and the Hazard of Discharge

AKI status	Hazard of discharge (increased LOS)					
	Crude HR (95% CI)	*P* value	Adjusted HR (95% CI)^a^^,^^b^	*P* value	Final adjusted HR (95% CI)^a^^,^^c^	*P* value
Overall						
No AKI	1 [Reference]	NA	1 [Reference]	NA	1 [Reference]	NA
sAKI	0.9 (0.8-1.0)	.01	0.8 (0.7-0.9)	<.001	0.8 (0.7-0.9)	.001
rAKI	0.6 (0.5-0.8)	<.001	0.5 (0.4-0.7)	<.001	0.6 (0.5-0.8)	<.001
rAKI vs sAKI	0.7 (0.6-0.9)	.002	0.7 (0.5-0.9)	.001	0.7 (0.6-0.9)	.01
Gestational age <29 wk						
No AKI	1 [Reference]	NA	1 [Reference]	NA	1 [Reference]	NA
sAKI	1.0 (0.7-1.3)	.83	1.0 (0.8-1.4)	.80	1.0 (0.8-1.4)	.83
rAKI	0.9 (0.6-1.3)	.44	1.0 (0.7-1.5)	.99	1.0 (0.6-1.5)	.82
rAKI vs sAKI	0.9 (0.6-1.3)	.55	1.0 (0.6-1.5)	.87	0.9 (0.6-1.4)	.70
Gestational age 29 to <36 wk						
No AKI	1 [Reference]	NA	1 [Reference]	NA	1 [Reference]	NA
sAKI	0.8 (0.7-1.0)	.08	0.8 (0.6-0.9)	.008	0.8 (0.6-0.9)	.007
rAKI	0.6 (0.4-0.9)	.02	0.6 (0.4-1.0)	.04	0.6 (0.4-1.0)	.05
rAKI vs sAKI	0.7 (0.4-1.1)	.14	0.8 (0.5-1.3)	.42	0.9 (0.5-1.4)	.50
Gestational age ≥36 wk						
No AKI	1 [Reference]	NA	1 [Reference]	NA	1 [Reference]	NA
sAKI	0.8 (0.7-0.9)	.002	0.8 (0.6-0.9)	.001	0.8 (0.7-1.0)	.01
rAKI	0.4 (0.3-0.6)	<.001	0.4 (0.3-0.5)	<.001	0.5 (0.3-0.7)	<.001
rAKI vs sAKI	0.5 (0.4-0.7)	<.001	0.5 (0.4-0.7)	<.001	0.6 (0.4-0.8)	.002
*P* value for gestational age interaction	NA	.07	NA	.005	NA	.07

Abbreviations: AKI, acute kidney injury; LOS, length of stay; NA, not applicable; rAKI, recurrent AKI; sAKI, single AKI.

^a^ Estimated from a time-varying Cox proportional hazards regression stratified by study site to account for potential clustering.

^b^ Adjusted for gestational age, birthweight, and 5-minute Apgar score.

^c^ Adjusted for gestational age (unless stratified by gestational age), race and ethnicity, birthweight, 5-minute Apgar score, admission for respiratory symptoms, admission for congenital heart disease, admission for surgical evaluation, preeclampsia, intrauterine growth restriction, antenatal corticosteroid use, meconium-stained fluid, and amniotic fluid level.

### rAKI Outcomes: Associations With Mortality and Hazard of Death

Due to the potential for a change in AKI status over time affecting associations with mortality, Cox proportional hazards regression modeling was used (Table 4). The eFigure in Supplement 1 depicts survival by AKI status. Among the overall cohort after adjusting for multiple potential confounders in the best-fit final model, sAKI (AHR, 3.6 [95% CI, 2.0-6.4]; *P* < .001) and rAKI (AHR, 4.9 [95% CI, 2.0-12.0]; *P* < .001) remained associated with an increased hazard of death when compared with no AKI. However, there was no significant difference in mortality when comparing rAKI with sAKI.

**Table 4.** Associations Between Time-Varying AKI Status and Mortality

AKI status	Hazard of mortality						
	Crude HR (95% CI)	*P* value	Adjusted HR (95% CI)^a^^,^^b^	*P* value	Final adjusted HR (95% CI)^a^^,^^c^	*P* value
Overall						
No AKI	1 [Reference]	NA	1 [Reference]	NA	1 [Reference]	NA
sAKI	5.0 (3.0-8.4)	<.001	3.8 (2.2-6.6)	<.001	3.6 (2.0-6.4)	<.001
rAKI	8.9 (4.0-19.9)	<.001	5.3 (2.3-12.3)	<.001	4.9 (2.0-12.0)	<.001
rAKI vs sAKI	1.8 (0.9-3.7)	.13	1.4 (0.7-3.0)	.37	1.4 (0.6-3.0)	.44
Gestational age <29 wk						
No AKI	1 [Reference]	NA	1 [Reference]	NA	1 [Reference]	NA
sAKI	3.7 (1.8-7.8)	<.001	2.97 (1.4-6.3)	.005	3.1 (1.4-6.7)	.004
rAKI	5.6 (2.0-15.2)	<.001	3.6 (1.3-10.3)	.02	3.4 (1.1-10.6)	.03
rAKI vs sAKI	1.5 (0.6-3.8)	.41	1.2 (0.5-3.3)	.69	1.1 (0.4-3.2)	.86
Gestational age 29 to <36 wk						
No AKI	1 [Reference]	NA	1 [Reference]	NA	1 [Reference]	NA
sAKI	13.9 (5.2-37.6)	<.001	10.8 (3.6-32.3)	<.001	10.4 (3.3-33.0)	<.001
rAKI	7.9 (0.8-75.7)	.07	6.9 (0.7-70.4)	.11	6.8 (0.6-75.3)	.12
rAKI vs sAKI	0.6 (0.1-5.0)	.61	0.6 (0.1-6.0)	.69	0.7 (0.1-6.5)	.72
Gestational age ≥36 wk						
No AKI	1 [Reference]	NA	1 [Reference]	NA	1 [Reference]	NA
sAKI	2.3 (0.9-6.0)	.10	2.4 (0.8-6.6)	.11	2.1 (0.8-6.0)	.15
rAKI	9.1 (2.7-31.4)	<.001	7.6 (2.1-27.7)	.002	6.9 (1.9-25.7)	.004
rAKI vs sAKI	4.1 (1.2-14.2)	.03	3.3 (0.9-11.5)	.07	3.3 (0.9-11.7)	.07
*P* value for gestational age interaction	NA	.06	NA	.16	NA	.19

Abbreviations: AKI, acute kidney injury; NA, not applicable; rAKI, recurrent AKI; sAKI, single AKI.

^a^ Estimated from a time-varying Cox proportional hazards regression stratified by study site to account for potential clustering.

^b^ Adjusted for gestational age, birthweight, and 5-minute Apgar score.

^c^ Adjusted for gestational age (unless stratified by gestational age, race and ethnicity, birthweight 5-minute Apgar score, admission for prematurity, admission for respiratory failure, intrauterine growth restriction, and maternal age).

Similar to LOS, significant differences were noted when examining associations between AKI status and mortality by gestational age. While multiple instances of increased hazard of death were detected in each gestational age strata between those with sAKI or rAKI compared with those without AKI, we did not detect any increased hazard of death with rAKI compared with sAKI (Table 4). In those with gestational age of at least 36 weeks, only rAKI was associated with a significant hazard of death (AHR, 6.9 [95% CI, 1.9-25.7]; *P* = .004) when compared with those without AKI.

## Discussion

As the effect of AKI becomes clearer in critically ill neonates,^1,4,5,7,8^ it is important to better understand high-risk states such as rAKI, which has been understudied. To our knowledge, this secondary analysis of the AWAKEN study is the first multicenter analysis of the incidence, risk factors, and outcomes associated with rAKI in a broad cohort of critically ill neonates. We show that rAKI in the NICU is common, with 22.0% of the neonates with AKI in the AWAKEN cohort experiencing rAKI, and that rAKI is associated with adverse outcomes, specifically longer LOS when compared with neonates who experience sAKI.

To date, only 2 single-center studies^13,16^ have evaluated risk factors for rAKI in neonates. We confirm the previous findings by identifying younger gestational age, lower birthweight, and a more severe initial AKI episode as risk factors for rAKI. These findings should raise awareness for these high-risk populations meriting increased surveillance. Increased education and dissemination of consensus guidelines for quality post-AKI care may lead to earlier implementation to prevent or diagnose rAKI sooner in hopes of improving long-term kidney health.^19^

The findings of the present study suggest that neonates with rAKI had a significantly lower hazard of discharge than those with sAKI and experienced substantially longer LOS. We were unable to determine whether rAKI was independently associated with longer LOS, or rather, whether infants with longer LOS experienced more potential days in the NICU to be at risk for rAKI; however, the time-varying Cox proportional hazards regression model provides more plausibility for the former. To better understand this association between rAKI and LOS, we stratified it by gestational age and found that it was only present in neonates with gestational age of at least 36 weeks; no associations were found in neonates with gestational ages of less than 29 weeks or 29 to less than 36 weeks. One potential explanation for this may be that premature infants typically have longer LOS compared with infants with older gestational age, regardless of AKI status. Future studies are needed to better understand the relationship between rAKI and LOS; an association in either direction is important to know for this population, as there is an opportunity to intervene and prevent an infant with sAKI from developing rAKI.

Among the overall cohort, infants with rAKI did not experience an increased hazard of death compared with those with sAKI; however, only rAKI was associated with increased hazard of death when examining infants at a gestational age of 36 weeks or more, after adjusting for multiple potential confounders. The differential presence of an association between rAKI and mortality among those with gestational age of 36 weeks or more could be explained by premature infants being more likely to succumb to disease processes that can be associated with both AKI and mortality (ie, necrotizing enterocolitis),^29,30,31^ whereas more mature neonates are likely to survive their first AKI episode, with no association found between sAKI and mortality in the group with gestational age of 36 weeks or more.

Interestingly, when comparing rAKI and sAKI, our findings regarding associations with mortality when accounting for time-varying AKI status differed from those of both prior single-center studies.^13,16^ Notably, AWAKEN was a larger, multicenter cohort, and these differences could be explained by variation in distribution of gestational age among the cohorts highlighted by distinct gestational age group interactions not detected in the prior studies. Similar to rAKI and LOS, the lack of an association between rAKI and mortality when compared with sAKI could reflect immortal time or survival bias, given that a patient has to survive long enough to develop rAKI. Surviving allows for more opportunity to encounter nephrotoxic exposures, for example, and for rAKI to occur. Any association between rAKI and mortality is also confounded by the competing risk of death and the inability to develop rAKI. These results should be interpreted with caution, as there was an overall low mortality rate in the study.

Our improved understanding of the incidence and impact of neonatal AKI has been driven in large part by the adoption of a consensus definition of AKI.^32,33^ Since that adoption, opportunities for improvement in the modified, neonatal KDIGO definition have been identified, including but not limited to gestational age–varying SCr thresholds, failure of SCr level to decline after birth, fluid-corrected SCr level, and ideal UOP criteria.^34,35,36^ We acknowledge that ideal criteria to differentiate between continuation of an initial AKI episode vs a new (and thus recurrent) AKI episode should be further elucidated, and the determination of an optimal and standardized definition of rAKI is still needed. The present study raises important questions about rAKI, such as the timing of greatest risk and the importance of duration of AKI, which may help signify the impact of injury. We also recognize rAKI as a high-risk state not just limited to neonates, as Gubb et al^37^ demonstrated increased 30-day mortality and prolonged kidney impairment in pediatric patients with rAKI. Although the long-term implications for neonatal survivors of rAKI are not yet known, rAKI is likely an important indicator that may be used to identify those at increased risk for chronic kidney disease requiring long-term follow up.

### Limitations

This study has some limitations. Although time-varying Cox proportional hazards regression models were used to address potential sources of bias (ie, immortal time and survival bias), a limitation of the dataset is that all potential covariates collected were maternal or neonatal characteristics that were determined at birth and unchanging (ie, time-invariant), and therefore our models do not account for the potential for time-varying confounding from other possible explanatory variables. Retrospective analysis of neonatal AKI using KDIGO criteria depends on the frequency of SCr measurements and the availability and reliability of UOP data. In addition, we used a conservative definition of rAKI requiring a complete return to baseline SCr level to distinguish rAKI from continuation of the prior AKI episode, which may underestimate the incidence of rAKI. Given increased research activity on neonatal kidney disease, institutions are likely monitoring SCr concentrations more closely at present. Increased awareness may result in a higher incidence of recognized AKI and perhaps a lower recurrence rate if recognition prompts earlier implementation of kidney-protective strategies post AKI.

## Conclusions

In this multicenter cohort study of 2162 neonates, we show that rAKI occurs commonly and is independently associated with adverse outcomes, specifically longer LOS. These results support the paradigm that rAKI is likely an important and distinct clinical entity meriting increased surveillance after an initial AKI episode. The present study provides important information that will inform the development of evidence-based post-AKI care guidelines to prevent and diagnose rAKI sooner to improve outcomes. Furthermore, the present study suggests that rAKI should be independently studied as a contributor to long-term kidney health in neonates.

## Notes

Supplement 1. **eFigure.** Survival by Acute Kidney Injury Status

Supplement 2.
Nonauthor Collaborators

Supplement 3.
Data Sharing Statement
