# Supplementary material for: Incidence, Risk Factors, and Outcomes Associated With Recurrent Neonatal Acute Kidney Injury in the AWAKEN Study
Source: JAMA Netw Open. 2024 Feb 8;7(2):e2355307. doi: 10.1001/jamanetworkopen.2023.55307 (PMC10853837; doi:10.1001/jamanetworkopen.2023.55307)
Supplement: Supplement 1. — eFigure. Survival by Acute Kidney Injury Status [file jamanetwopen-e2355307-s001.pdf]

Supplementary Online Content

Rutledge AD, Griffin RL, Vincent K, et al; Neonatal Kidney Collaborative. Incidence, risk factors, and outcomes associated with recurrent neonatal acute kidney injury. *JAMA Netw Open*. 2024;7(2):e2355307. doi:10.1001/jamanetworkopen.2023.55307

eFigure. Survival by Acute Kidney Injury Status

This supplementary material has been provided by the authors to give readers additional information about their work.

© 2024 Rutledge AD et al. *JAMA Network Open*.

eFigure. Survival by Acute Kidney Injury Status

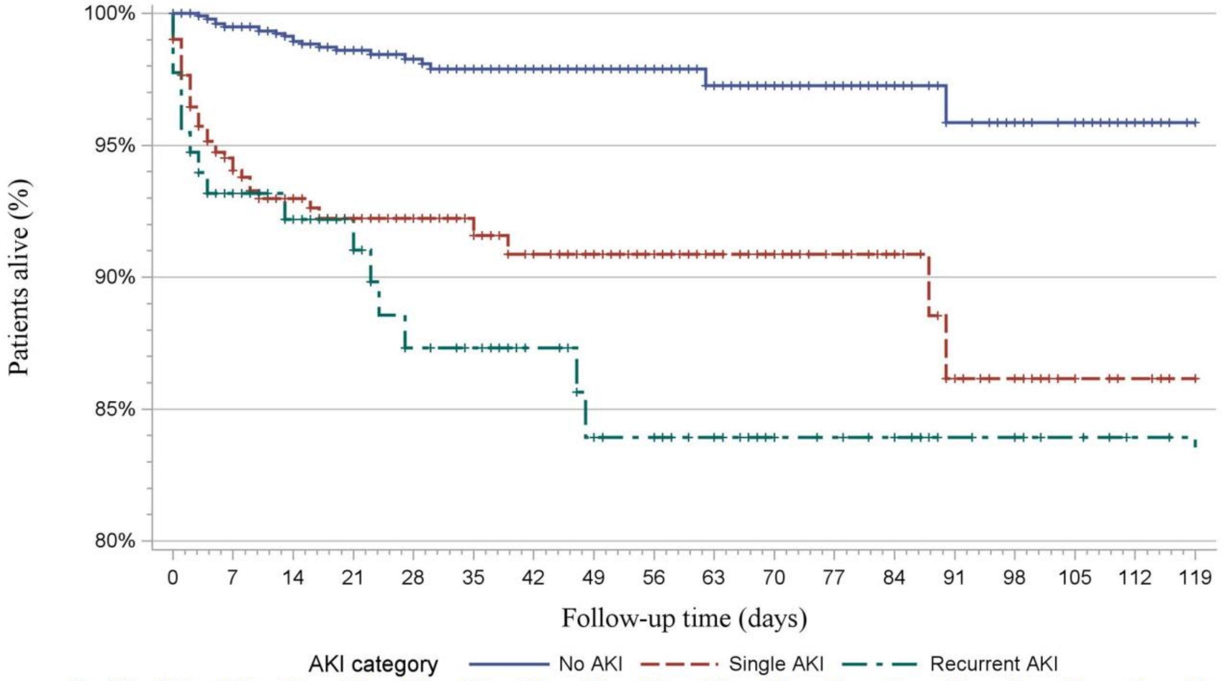

No AKI	2162	1461	990	718	545	406	314	250	196	145	115	97	82	66	57	51	41	31
Single AKI	605	399	286	199	163	141	124	106	87	70	62	50	43	35	29	21	16	12
Recurrent AKI	133	109	93	79	69	65	56	49	47	41	31	28	25	19	18	13	10	9

© 2024 Rutledge AD et al. *JAMA Network Open*.
