# Supplementary material for: Incidence, Risk Factors, and Outcomes Associated With Recurrent Neonatal Acute Kidney Injury in the AWAKEN Study
Source: JAMA Netw Open. 2024 Feb 8;7(2):e2355307. doi: 10.1001/jamanetworkopen.2023.55307 (PMC10853837; doi:10.1001/jamanetworkopen.2023.55307)
Supplement: Supplement 2. — Nonauthor Collaborators [file jamanetwopen-e2355307-s002.pdf]

Supplemental Online Content: Nonauthor Collaborators
*First name, last name, and suffix (if applicable) are required and will appear in PubMed.

*Group Name(s): Neonatal Kidney Collaborative							
*First Name and Middle Initial(s)	*Last Name	*Suffix	Academic Degrees	Institution	Location (city, state/province, country)	Role or Contribution, eg, chair, principal investigator	Group (if more than 1 Group listed in the byline) and/or Subgroup (eg, Steering Committee)
Subrata	Sarkar			CS Mott Children's Hospital, University of Michigan	Ann Arbor, MI, USA		
Alison	Kent			Centenary Hospital for Women and Children, Canberra Hospital, Australian National University Medical School<br><br>Golisano Children's Hospital, University of Rochester	Canberra, ACT, Australia<br><br>Rochester, NY, USA		
Jeffery	Fletcher			Centenary Hospital for Women and Children, Canberra Hospital, Australian National University Medical School	Canberra, ACT, Australia		
Jennifer R.	Charlton			University of Virginia Children's Hospital	Charlottesville, VA, USA		
Jonathan R.	Swanson			University of Virginia Children's Hospital	Charlottesville, VA, USA		
Carolyn L.	Abitbol			Holtz Children's Hospital, University of Miami,	Miami, FL, USA		
Marissa	DeFreitas			Holtz Children's Hospital, University of Miami,	Miami, FL, USA		
Shahnaz	Duara			Holtz Children's Hospital, University of Miami,	Miami, FL, USA		
Ronnie	Guillet			Golisano Children's Hospital, University of Rochester	Rochester, NY, USA		
Carl	D'Angio			Golisano Children's Hospital, University of Rochester	Rochester, NY, USA		
Ayesa	Mian			Golisano Children's Hospital, University of Rochester	Rochester, NY, USA		
Erin	Rademacher			Golisano Children's Hospital, University of Rochester	Rochester, NY, USA		
Maroun J.	Mhanna			MetroHealth Medical Center, Case Western Reserve University<br><br>Ochsner/Louisiana State University Health	Cleveland, OH, USA<br><br>Shreveport, LA, USA		
Rupesh	Raina			MetroHealth Medical Center, Case Western Reserve University	Cleveland, OH, USA		
Deepak	Kumar			MetroHealth Medical Center, Case Western Reserve University	Cleveland, OH, USA		
Namasivayam	Ambalavanan			University of Alabama at Birmingham	Birmingham, AL, USA		
Ayse A.	Arikan			Texas Children's Hospital, Baylor College of Medicine	Houston, TX, USA		
Christopher J.	Rhee			Texas Children's Hospital, Baylor College of Medicine	Houston, TX, USA		
Stuart L.	Goldstein			Cincinnati Children's Hospital and Medical Center, University of Cincinnati	Cincinnati, OH, USA		
Amy T.	Nathan			Cincinnati Children's Hospital and Medical Center, University of Cincinnati	Cincinnati, OH, USA		
Alok	Bhutada			Maimonides Medical Center	Brooklyn, NY, USA		
Elizabeth	Bonachea			Nationwide Children's Hospital	Columbus, OH, USA		
Susan	Ingraham			Nationwide Children's Hospital<br><br>Kapi'olani Medical Center for Women & Children	Columbus, OH, USA<br><br>Honolulu, HI, USA		
John	Mahan			Nationwide Children's Hospital	Columbus, OH, USA		
Arwa	Nada			Nationwide Children's Hospital<br><br>LeBonheur's Children's Hospital/University of Tennessee	Columbus, OH, USA<br><br>Memphis, TN, USA		
Jennifer	Jetton			University of Iowa Children's Hospital	Iowa City, IA, USA		
Patrick D.	Brophy			University of Iowa Children's Hospital<br><br>Golisano Children's Hospital, University of Rochester	Iowa City, IA, USA<br><br>Rochester, NY, USA		
Tarah T.	Colaizy			University of Iowa Children's Hospital	Iowa City, IA, USA		
Jonathan M.	Klein			University of Iowa Children's Hospital	Iowa City, IA, USA		
F. Sessions	Cole			Washington University	St Louis, MO, USA		
T. Keefe	Davis			Washington University<br><br>University of Saskatchewan	St Louis, MO, USA<br><br>Saskatoon, SK, Canada		
Joshua	Dower			Tufts University School of Medicine	Boston, MA, USA		
Lawrence	Milner			Tufts University School of Medicine	Boston, MA, USA		
Alexandra	Smith			Tufts University School of Medicine	Boston, MA, USA		
Mamta	Fuloria			The Children's Hospital at Montefiore	Bronx, NY, USA		
Kimberly	Reidy			The Children's Hospital at Montefiore	Bronx, NY, USA		
Frederick J.	Kaskel			The Children's Hospital at Montefiore	Bronx, NY, USA		
Danielle E.	Soranno			University of Colorado, Children's Hospital Colorado<br><br>Indiana University School of Medicine	Aurora, CO, USA<br><br>Indianapolis, IN, USA		
Jason	Gien			University of Colorado, Children's Hospital Colorado	Aurora, CO, USA		
Katja M.	Gist			University of Colorado, Children's Hospital Colorado	Aurora, CO, USA		
Mina H.	Hanna			University of Kentucky	Lexington, KY, USA		

Supplemental Online Content: Nonauthor Collaborators
*First name, last name, and suffix (if applicable) are required and will appear in PubMed.

***First Name and Middle Initial(s)**	***Last Name**	***Suffix**	Academic Degrees	Institution	Location (city, state/province, country)	Role or Contribution, eg, chair, principal investigator	Group (if more than 1 Group listed in the byline) and/or Subgroup (eg, Steering Committee)
Aftab S.	Chishti			University of Kentucky	Lexington, KY, USA		
Sangeeta	Hingorani			University of Washington, Seattle Children's Hospital	Seattle, WA, USA		
Sandra	Juul			University of Washington, Seattle Children's Hospital	Seattle, WA, USA		
Michelle C.	Starr			Indiana University School of Medicine<br><br>University of Washington, Seattle Children's Hospital	Indianapolis, IN, USA<br><br>Seattle, WA, USA		
Craig S.	Wong			University of New Mexico Health Sciences Center	Albuquerque, NM, USA		
Catherine	Joseph			University of New Mexico Health Sciences Center	Albuquerque, NM, USA		
Tara	DuPont			University of New Mexico Health Sciences Center	Albuquerque, NM, USA		
Robin	Ohls			University of New Mexico Health Sciences Center<br><br>University of Utah	Albuquerque, NM, USA<br><br>Salt Lake City, UT, USA		
Amy	Staples			University of New Mexico Health Sciences Center	Albuquerque, NM, USA		
Smriti	Rohatgi			Kidney and Urology Institute Medanta—The Medicity	Gurgaon, India		
Sidharth K.	Sethi			Kidney and Urology Institute Medanta—The Medicity	Gurgaon, India		
Sanjay	Wazir			Cloudnine Hospital	Gurgaon, Haryana, India		
Surender	Khokhar			Apollo Cradle	Gurgaon, Haryana, India		
Sofia	Perazzo			Children's National Medical Center, George Washington University School of Medicine and the Health Sciences	Washington, DC, USA		
Patricio E.	Ray			Children's National Medical Center, George Washington University School of Medicine and the Health Sciences<br><br>University of Virginia Children's Hospital,	Washington, DC, USA<br><br>Charlottesville, VA, USA		
Mary	Revenis			Children's National Medical Center, George Washington University School of Medicine and the Health Sciences	Washington, DC, USA		
Cherry	Mammen			British Columbia Children's Hospital	Vancouver, BC, Canada		
Anne	Synnes			British Columbia Children's Hospital	Vancouver, BC, Canada		
Pia	Wintermark			Montreal Children's Hospital, McGill University Health Centre	Montreal, QC, Canada		
Michael	Zappitelli			Montreal Children's Hospital, McGill University Health Centre<br><br>The Hospital for Sick Children	Montreal, QC, Canada<br><br>Toronto, ON, Canada		
Robert	Woroniecki			Stony Brook School of Medicine	Stony Brook, NY, USA		
Shanthy	Sridhar			Stony Brook School of Medicine	Stony Brook, NY, USA		
