# Supplementary material for: Incidence, Risk Factors, and Outcomes Associated With Recurrent Neonatal Acute Kidney Injury in the AWAKEN Study
Source: JAMA Netw Open. 2024 Feb 8;7(2):e2355307. doi: 10.1001/jamanetworkopen.2023.55307 (PMC10853837; doi:10.1001/jamanetworkopen.2023.55307)
Supplement: Supplement 3. — Data Sharing Statement [file jamanetwopen-e2355307-s003.pdf]

Data Sharing Statement

Rutledge. Incidence, Risk Factors, and Outcomes Associated With Recurrent Neonatal Acute Kidney Injury in the AWAKEN Study. *JAMA Netw Open*. Published February 08, 2024. doi:10.1001/jamanetworkopen.2023.55307

Data
Data available: No
